# Laparoscopic transabdominal preperitoneal herniorrhaphy performed using an articulating laparoscopic instrument is feasible and more efficient

**DOI:** 10.3389/fsurg.2023.1305320

**Published:** 2024-01-04

**Authors:** Jung Hyun Park, Dong Jin Kim

**Affiliations:** Department of Surgery, College of Medicine, The Catholic University of Korea, Seoul, Republic of Korea

**Keywords:** laparoscopy, inguinal hernia, ergonomic, articulating instrument, learning curve

## Abstract

**Introduction:**

Ipsilateral left-sided-approach laparoscopic transabdominal preperitoneal herniorrhaphy (LA-TAPP) is a procedure used for inguinal hernia. However, conventional laparoscopic instruments may limit the operator's ability to approach certain areas during the procedure. This study aims to assess the feasibility of using an articulating bipolar grasper (ArtiSential®).

**Material and methods:**

Between January 2017 and May 2022, 184 patients with inguinal hernia underwent LA-TAPP and were divided into an articulating group (AG) and a conventional group (CG). The two groups were compared for clinical characteristics, surgical outcomes, and recurrence rates. Learning curve analysis was also performed using the CUSUM score.

**Results:**

The AG and CG included 72 and 112 patients, respectively. Both groups had similar age, sex, BMI, hernia location, and hernia type. The AG had a significantly shorter operation time (59.2 ± 29.4 vs. 77.8 ± 22.4 min, *p* < 0.001) than the CG. The duration of hospitalization was slightly shorter in the AG (2.2 ± 0.5 vs. 2.5 ± 1.4 days, *p* = 0.056). Postoperative complications were lower in the AG (5.6%) than in the CG (9.8%). Scrotal neuralgic pain was observed in 1.4% of patients in the AG and 3.6% of patients in the CG. Learning curve analysis revealed that 24 cases were needed to overcome the learning curve for using an articulating device.

**Conclusion:**

IP-TAPP with an articulating instrument is a safe and efficient procedure. The operation time can be reduced by improving the surgeon's procedural autonomy and reducing collisions between the instruments and the patient's ribs.

## Introduction

Inguinal hernia is one of the most frequently encountered conditions requiring surgery. Since the first endoscopic inguinal hernia repair was reported in 1991, laparoscopic inguinal hernia repair has been increasingly adopted owing to its advantages such as less pain and early recovery (1–4). The two representative procedures for laparoscopic inguinal herniorrhaphy are the totally extraperitoneal (TEP) method and the transabdominal preperitoneal (TAPP) method (1, 5). The selection of the optimal procedure for laparoscopic herniorrhaphy has been an extensively debated topic with no consensus. Although both procedures have several advantages and disadvantages, TAPP seems easy to learn, technically simple, and provides a better anatomical view. In addition, it can also be adopted for patients with a history of lower abdominal or pelvic surgery and recurrent inguinal hernia after TEP.

During the TAPP procedure, contralateral trocar placement was usually performed for the surgeons' right- and left-hand instruments. Since this placement approach results in a non-ergonomic trocar location for surgeons with a short arm reach, our surgical team attempted to employ only left-sided trocar placement for bilateral inguinal hernia (6). The findings indicated the feasibility of a left-side-only approach for bilateral inguinal hernias in comparison with the TEP procedure. Therefore, we introduced the left-side approach TAPP (LA-TAPP) procedure. In this procedure, we used umbilical trocar as optical scope and operator's right and left hand instruments are introduced through patients' left side only regardless of hernia location. In LA-TAPP, preperitoneal dissection on the left lateral and right medial sides was difficult to perform with conventional instrument not like with conventional contra-lateral trocar position. In some patients, the rib cage is lower than usual, causing frequent collisions between the right-hand instrument and the patient's lower rib margin. To solve these problems, we used the Artisential® (LIVSMED Inc., Republic of Korea) bipolar grasper, a new laparoscopic articulating instrument, to dissect the preperitoneal space during laparoscopic LA-TAPP. This study aimed to determine the feasibility and efficiency of using Artisential during the TAPP procedure.

## Materials and methods

We included patients who underwent laparoscopic LA-TAPP performed by a single surgeon between January 2017 and May 2022 at a single institution. The data were collected retrospectively. The patients were divided into articulating (AG) and conventional (CG) groups. The clinical characteristics, surgical results, and long-term recurrences were compared between the two groups.

Patient characteristics included age, sex, body mass index (BMI), hernia type and location, and recurrent hernia status. Operative time, postoperative complications, and early recurrence were documented. Additionally, learning curve analysis for both procedures was performed using CUSUM analysis. This study was approved by the Institutional Ethical Review Board (IRB number: PC17REDI0055), and the authors have no conflicts of interest to disclose.

## Operative procedure

**Figure 1:**
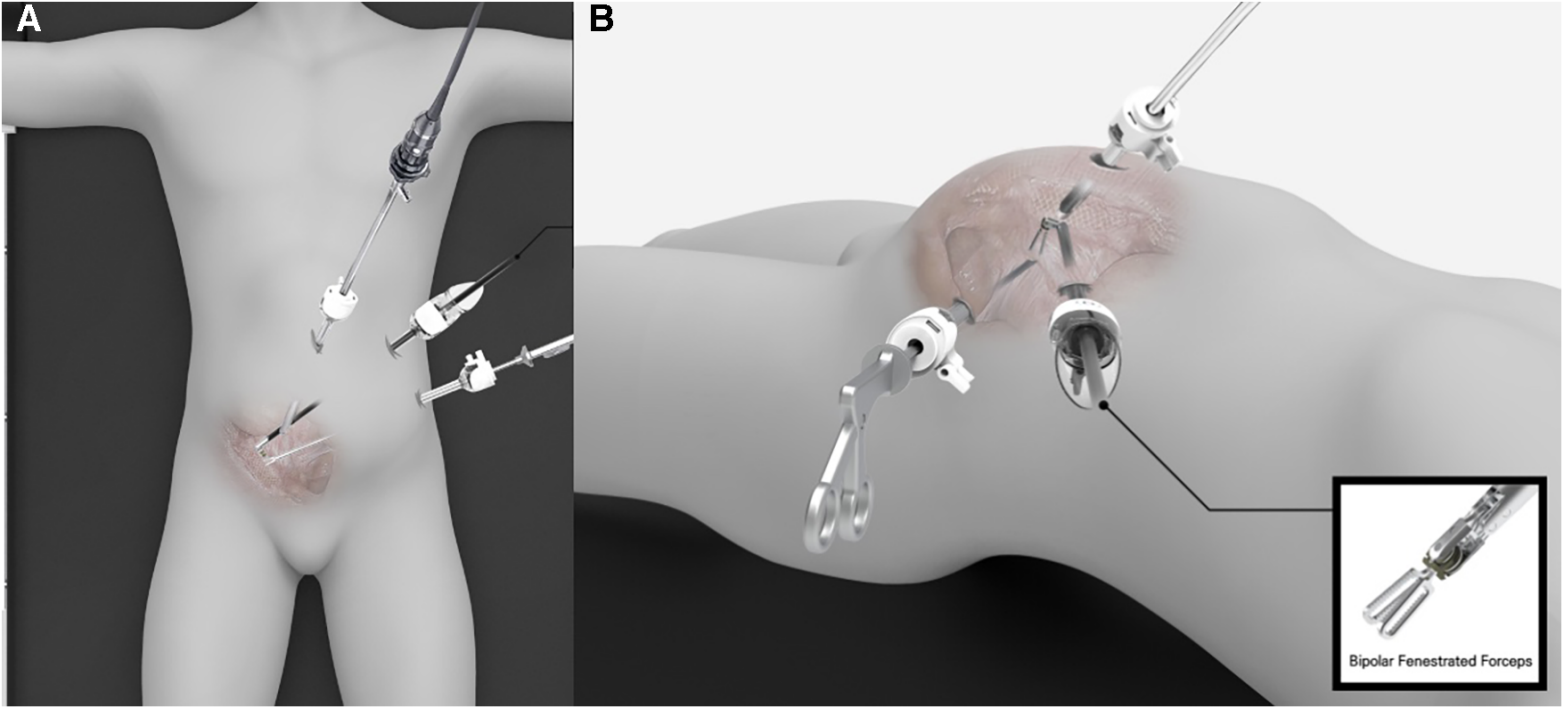
Trocar placement for ipsilateral trans-abdominal preperitoneal herniorrhaphy using laparoscopic articulating instruments. (**A**) Up-down view, (**B**) Left lateral view.

The operative procedure for the CG was the same as that reported previously (6). The LA-TAPP using articulation was performed using the same trocar placement as in the CG. However, a right-hand trocar with a diameter of 8 mm was used. Thus, the trocar placements were as follows: a 10 mm optical trocar at the umbilicus, a 5 mm operative trocar at the midaxillary line 4–5 cm upward from the anterior superior iliac spine (ASIS), and an 8 mm operative trocar at the midaxillary line 5 cm in a cephalic direction from the previous operative trocar (Figure 1).

## Operative procedure using an articulating instrument

**Figure 2:**
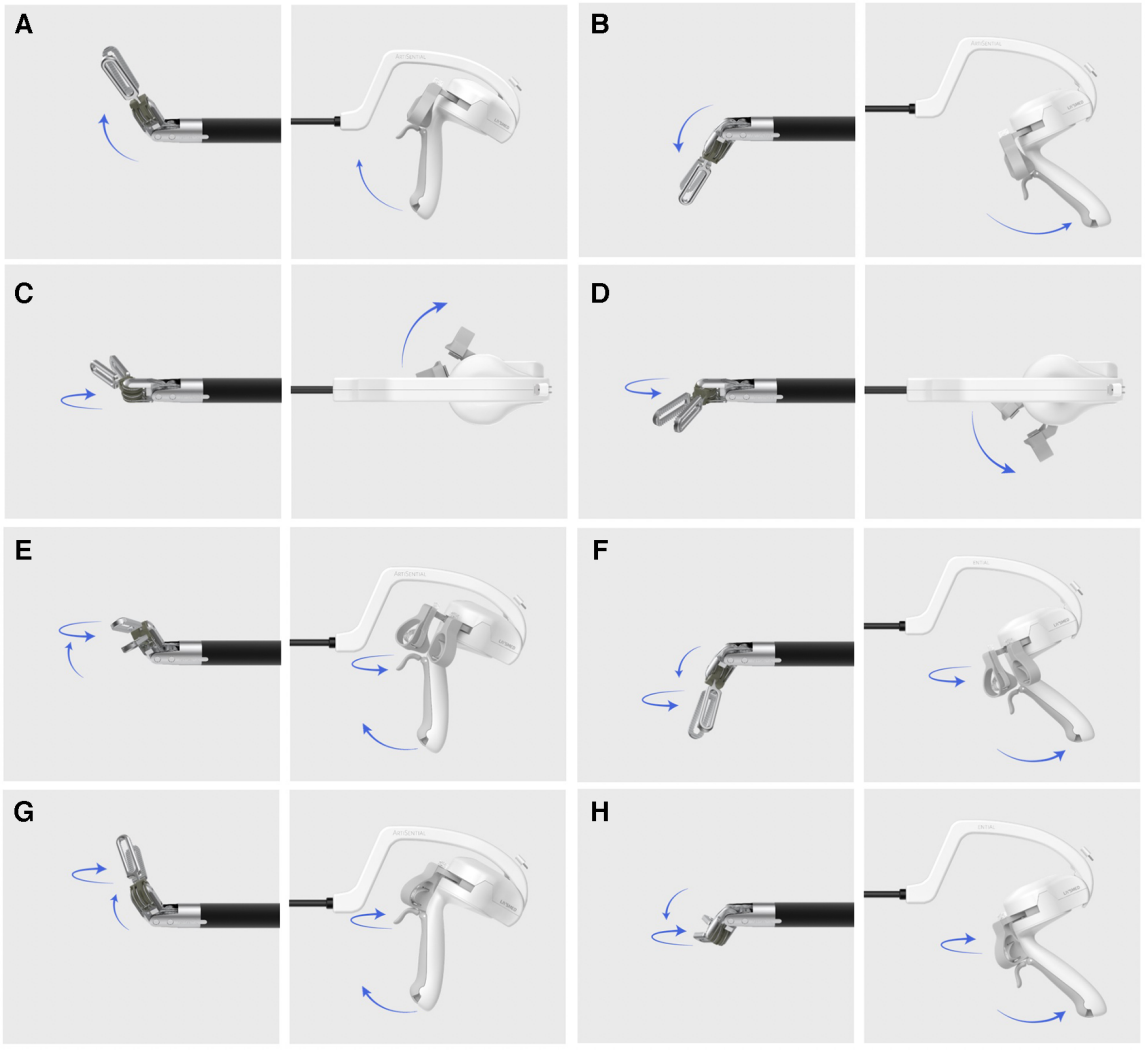
Movement of articulating instrument. (**A**) Up, (**B**) Down, (**C**) Right, (**D**) Left, (**E**) Upper-left, (**F**) Lower-left, (**G**) Upper right, (**H**) Lower right.

After the peritoneum was incised in an up-to-down direction with a long straight electrocautery device, preperitoneal dissection was initiated with a right-hand bipolar articulating device. Movement mechanism is described at Figure 2. Tip of instrument can be articulated every direction up to 90 degree according to the movement of surgeons' wrist. Repeated blunt dissection and cauterization of the vessel-containing tissue with bipolar energy were performed until the symphysis pubis was exposed medially and the ASIS was exposed on the lateral side. In cases involving indirect inguinal hernia, after identifying the hernial sac and cord structure entering the internal ring, the hernial sac was isolated from the spermatic cord and vessels using an articulating grasper. During sac dissection, minor vessels were cauterized in coagulation mode, and thick adhesive lesions were divided using the cutting mode of the bipolar instrument.

After preperitoneal dissection and hernia sac peritonealization, a 3D lightweight polypropylene mesh was introduced through the 8-mm trocar. After placing the mesh in the correct position, fibrin glue was applied to the lower border of the mesh. Fibrin glue was applied with laparoscopic long fibrin glue applier. During the early period of LA-TAPP, mesh fixation was performed using a capsule at Cooper's ligament and the transverse abdominis muscle near the ASIS. The peritoneum was then closed using barbed suture material. During the peritoneum closure. We used conventional needle holder instrument. With the current trocar placements, a suturing the incised peritoneum is not difficult with the conventional instrument. Procedure was briefly edited in video file (Supplementary Video S1).

## Statistical analysis

Statistical analyses were performed with SPSS 18.0 (IBM, Chicago, IL, USA). The collected data were expressed as median, frequency, percentage, and mean ± standard deviation (SD). Chi-square test, Fisher's exact test, and Student's *t*-test were used for comparisons. CUSUM model analysis was performed using R statistics. Statistical significance was set at *p* < 0.05.

## Results

Among the 184 patients who underwent IP-TAPP, 72 and 112 belonged to the AG and CG, respectively (Table 1), including three (4.2%) and six (5.4%) female patients in the AG and CG, respectively. Mean patient age was 63.9 ± 15.8 years in the AG and 67.2 ± 14.0 years in the CG (*p* = 0.139). The mean BMI was similar in the two groups (AG vs. CG = 23.5 ± 3.0 vs. 23.6 ± 2.9, *p* = 0.893). Both groups had a similar proportion of patients with a history of abdominal surgery (AG vs. CG = 23 patients [34.7%] vs. 32 patients [28.6%], *p* = 0.473). The location of inguinal hernia was similar in the two groups. Bilateral inguinal hernias were observed in 6.9% of the cases in the AG and 10.7% of those in the CG. Seven (9.7%) and eight (7.1%) patients in the AG and CG had recurrent hernias, respectively.

**Table 1:** Clinical characteristics between articulating group and conventional group.

Variables	Articulating group	Conventional group	*P*-value
	(*N* = 72)	(*N* = 112)	
Age (year)	63.9 ± 15.8	67.2 ± 14.0	0.139
Sex			
Male	69 (95.8%)	106 (94.6%)	0.988
Female	3 (4.2%)	6 (5.4%)	
ASA			
1	30 (41.7%)	24 (21.4%)	0.013
2	35 (48.6%)	73 (65.2%)	
3	7 (9.7%)	15 (13.4%)	
BMI (kg/m^2^)	23.5 ± 3.0	23.6 ± 2.9	0.893
Location			
Bilateral	5 (6.9%)	12 (10.7%)	0.616
Right	38 (52.8%)	53 (47.3%)	
Left	29 (40.3%)	47 (42.0%)	
Type of Inguinal hernia			
Direct	23 (31.9%)	26 (23.2%)	0.256
Indirect	49 (68.1%)	86 (76.8%)	
Recurrent hernia			
Primary	65 (90.3%)	104 (92.9%)	0.728
Recured	7 (9.7%)	8 (7.1%)	
Previous abdominal operation history			
Present	25 (34.7%)	32 (28.6%)	0.473

Continuous variables were expressed as mean ± standard deviation, nominal variables were expressed as number (percentage).

The AG showed a significantly shorter operation time for all included cases and the subgroup of cases with unilateral inguinal hernias (Table 2). Mean operative time in all cases in the AG was 59.2 ± 29.4 min, and that in the CG was 77.8 ± 22.4 min (*p* = 0.0016). Among the subgroups with unilateral inguinal hernias, mean operation times were 57.2 ± 27.8 min and 75.7 ± 22.2 min in the AG and CG (*p* < 0.001), respectively. The mesh fixation method differed significantly between the two groups (*p* < 0.001). Hospital stay was shorter in the AG than in the CG (AG vs. CG = 2.2 ± 0.5 days vs. 2.5 ± 1.4 days, *p* = 0.05). The number of cases showing complications was 4 (5.6%) in the AG and 11 (9.8%) in the CG, but the difference was not significant. Postoperative seroma was the most frequent complication, and it occurred in two and five patients in the AG and CG, respectively. Chronic pain occurred in only one patient in the AG and three patients in the CG (*p* = 0.6). Both groups showed no early recurrence. However, one patient in the CG experienced recurrence 90 days after the initial operation.

**Table 2:** Surgical outcomes between articulating group and conventional group.

Variables	Articulating group	Conventional group	*P*-value
	(*N* = 72)	(*N* = 112)	
Op. Time_total case (min)	59.2 ± 29.4	77.8 ± 22.4	<0.0016
Op. Time_unilateral (min)	57.2 ± 27.8	75.7 ± 22.2	<0.001
Mesh fixation			
Tacker	45 (62.5%)	112 (100.0%)	<0.001
Glue	27 (37.5%)	0 (0.0%)	
Hospital stay (day)	2.2 ± 0.5	2.5 ± 1.4	0.05
Post operation complication	4 (5.6%)	11 (9.8%)	0.450
Seroma	2 (2.4%)	5 (4.5%)	
Chronic pain	1 (1.4%)	4 (3.6%)	
Hematoma	1 (1.4%)	1 (0.9%)	
Small bowel injury	-	1 (0.9%)	
Early recurrence (30 days)	0 (0%)	0 (0%)	-
Late recurrence	0 (0.0%)	1 (0.9%)	1.000
Mean follow up visit	1.8 ± 1.4	1.8 ± 1.7	0.725

Continuous variables were expressed as mean ± standard deviation, nominal variables were expressed as number (percentage).

**Figure 3:**
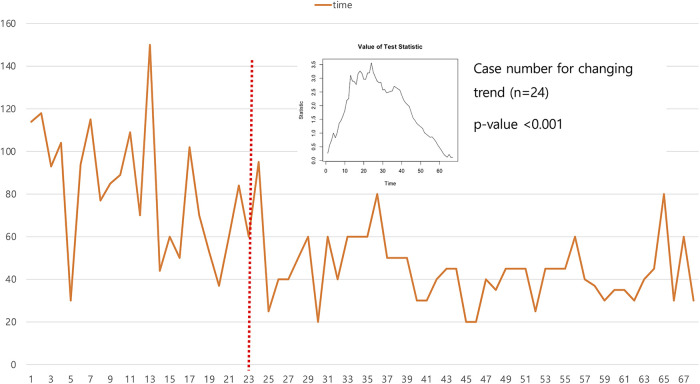
CUSUM analysis for operation time in articulating group showed 24 cases were needed to achieve the learning curve.

CUSUM analysis using unilateral patients revealed that the 25th case was the point at which the trend in operation time changed. The CUSUM score was 3.5 and the *p*-vale was less than 0.001 (Figure 3). However, we could not find a point corresponding to a significant shortening of the operation time.

## Discussion

This study demonstrated the usefulness of an articulating laparoscopic device for inguinal hernia surgery. Although we did not compare TAPP using an articulating device with TEP, one of the most frequently performed laparoscopic inguinal hernia surgeries, the articulating device yielded improvements in operative quality and time in comparison with LA-TAPP performed using a conventional instrument. The procedure performed with the articulating device required significantly less time without increasing the complication or recurrence rates. More importantly, these improvements were observed even though the surgeon was already experienced in performing the conventional procedure. Achieving sufficient surgical skill with the articulating device in terms of the operation time required 25 cases.

Laparoscopic hernia surgery shows various advantages over open inguinal hernia surgery (7–9), including lower operation times, early recovery, and fewer complications (10). However, the choice between TEP and TAPP remains unclear (11–13). For this reason, selection of the laparoscopic procedure is dependent on the surgeon's preference. The authors preferred TAPP because of its clear anatomic confirmation, which is feasible even in patients with a history of intra-abdominal operations such as prostatectomy or previous inguinal hernia surgery. Since the usual TAPP procedure uses contralateral trocars, it is difficult for a surgeon with a relatively short arm reach. Therefore, we developed a LA-TAPP (6). LA-TAPP is easy to perform and feasible for both right and left inguinal hernias. However, LA-TAPP using conventional laparoscopic instruments is associated with some obstacles. When the distance between the patient's umbilicus and the lower rib margin was small, the right-side instrument collided with the patient's left rib during peritoneal dissection and preperitoneal dissection of the most cephalic portion. Another problem is that the un-incised peritoneum from the midline to the median ligament interfered with preperitoneal dissection during TAPP for right inguinal hernia. The use of an articulating instrument resolved these issues. Moreover, the application of bipolar energy helped maintain a clear operational field. Another advantage of using this instrument is that we can minimize the peritoneal incision before pre-peritoneal dissection. Because articulating tip can dissect more space than conventional instrument can access. Regarding the suturing of the incised peritoneum, with current trocar placement, suturing is not difficult with conventional laparoscopic needle holder. During the suturing, the repeated procedure of rotating needle holder nearly 270 degrees and holding the needle tip with the left-hand instrument, adjusting the needle with the needle holder before tightening the barded suture material is an important tip.

Articulating devices have been introduced in several fields. Articulating graspers and monopolar devices have been used for colorectal cancer surgery (14, 15). An articulating needle holder has also been reported to be a useful instrument (16). To the best of our knowledge, this is the first study to demonstrate the usefulness of articulating laparoscopic instruments in inguinal hernia surgery.

The current study had some limitations. First, it was a retrospective study, and some bias may have been introduced because the use of the articulating instrument was started recently. However, the author had considerable experience with conventional IP-TAPP, and familiarization with the new instrument is expected to require some time. Second, we did not evaluate convenience or ergonomics in this study. Third limitation would be the accessibility of this instrument. This device is developed as single-use and costs nearly 500 USD.

## Conclusion

Ipsilateral TAPP with an articulating instrument is a safe and efficient procedure for treating inguinal hernias. Moreover, the operation time can be reduced by improving the surgeon's procedural autonomy and higher accessibility with minimal peritoneal incision. In addition, this procedure can reduce collisions between the instruments and the patient's rib.

## Data Availability

The datasets presented in this article are not readily available because The institutional review board of Eunpyeong St. Mary's Hospital does not permit the data supplement out of the institution. Requests to access the datasets should be directed to Dong Jin Kim, djdjcap@catholic.ac.kr.
